# Conversion of Methanol to Para-Xylene over ZSM-5 Zeolites Modified by Zinc and Phosphorus

**DOI:** 10.3390/molecules28134890

**Published:** 2023-06-21

**Authors:** Yang Bai, Xianjun Niu, Yi-En Du, Yongqiang Chen

**Affiliations:** 1 Department of Chemistry and Chemical Engineering, Jinzhong University, Jinzhong 030619, China; baiyang@jzxy.edu.cn (Y.B.); duyien124@163.com (Y.-E.D.); chenyongqiang82@126.com (Y.C.); 2 Department of Scientific Research, Jinzhong University, Jinzhong 030619, China

**Keywords:** methanol to aromatics, phosphorus source, Zn-P/ZSM-5, para-xylene

## Abstract

In this work, the influence of different phosphorus sources and the modification of zinc and phosphorus on the performance of the conversion of methanol to aromatics (MTA) was investigated. The results showed that the phosphorus source had a significant impact on the selectivity of para-xylene (PX) in xylene and catalyst stability. The introduction of P resulted in the covering of the active acid sites and the narrowing of the pore of the ZSM-5 zeolite, which improved the shape-selectivity for PX in the methanol conversion reaction. Compared with the modifiers of H_3_PO_4_ and (NH_4_)_3_PO_4_, the ZSM-5 zeolite modified by (NH_4_)_2_HPO_4_ exhibited better catalyst stability and PX-selectivity due to its larger specific surface area, pore volume and suitable acidity. When the ZSM-5 zeolite was modified by Zn and P, the effect of Zn and P on the selectivity to aromatics and PX in xylene was almost opposite. With the increase in P-loading, the selectivity of PX in xylene gradually increased but at the cost of decreasing the aromatic-selectivity. On the other hand, the loading of Zn introduced Zn-Lewis acid sites to provide aromatization active centers and improved the aromatic-selectivity. However, excessive Zn reduced the selectivity of PX in xylene. The catalyst activity and aromatic-selectivity could be improved to some extent with an appropriate ratio of Zn and P, while maintaining or increasing the para-selectivity of xylene. Compared with 5% P/ZSM-5 catalyst modified with only (NH_4_)_2_HPO_4_, the PX selectivity in xylene over the Zn-P/ZSM-5 catalyst modified with 5% Zn and 1% P improved from 86.6% to 90.1%, and the PX yield increased by 59%.

## 1. Introduction

Para-xylene (PX) is a significant organic chemical raw material used the production of terephthalic acid (PTA) or dimethyl terephthalate (DMT), which are widely utilized in various chemical production fields such as synthetic fibers, synthetic resins, pesticides, pharmaceuticals, and plastics [1,2,3]. However, traditional PX production relies heavily on the catalytic reforming of oil and the pyrolysis of gasoline as primary raw materials, both of which depend on petroleum resources and involve high energy consumption. Recently, the conversion of methanol to aromatics (MTA) has attracted great attention due to its potential for deriving methanol from coal, natural gas, or biomass, thus eliminating dependence on petroleum resources [4,5,6,7]. The unique shape selection of zeolites makes them the preferred catalysts for MTA, especially ZSM-5 zeolite whose pore size is similar than the kinetic diameter of benzene derivatives. Moreover, the acidity of ZSM-5 zeolite can be adjusted in a wide range via the introduction of heteroatoms, and the adjustment of crystal size and morphology, and its shape selection can be further enhanced by the external surface acid passivation, shrinkage, pore size, and other methods; thus, ZSM-5 zeolite is considered to be the most attractive methanol aromatization catalyst [5].

The technology of MTA has also been extensively studied, particularly focusing on surface modification of the ZSM-5 zeolite catalyst with metal elements such as Zn [8,9,10,11], Ga [12,13,14,15], Ag [16,17], and La [18,19]. The introduction of metal elements not only changes the physical properties of a ZSM-5 zeolite such as the specific surface area and pore structure, but also modifies its acidity, which can inhibit the hydrogen transfer reaction, reduce the generation of alkanes, and thus increase the yield of aromatics [20]. However, the selectivity of PX in xylene (X) is not significantly improved by only loading metal on ZSM-5 zeolites without other modifications of pore size and surface acidity. Therefore, it is necessary to design and modify the catalyst to make the reaction in the direction conducive to the formation of PX. In addition, the Si and P elements are often used as modifiers to improve the selectivity of PX in toluene disproportionation and toluene methylation reactions [21,22,23,24]. The combination of Si or P elements with ZSM-5 modified by a metal element is expected to increase the selectivity of PX while improving the yield of aromatics.

Currently, the surface modification of the ZSM-5 zeolite catalyst for MTA has made some progress in the conversion of methanol to para-xylene, providing a new pathway to produce PX. Zhu et al. [25] prepared a Mg-Zn-Si-HZSM-5 catalyst and found that the silica deposition significantly enhanced the selectivity of PX in xylene and inhibited the formation of C_9_^+^ aromatics due to the narrowing of the pore mouth and the elimination of external acid sites. Further Zn-loading effectively improved the MTA activity by promoting the dehydrogenation of intermediates through increasing the density of internal Zn-Lewis acid sites. However, the stability of the catalyst was poor, mainly due to the micropore blockage caused by coke deposition at the strong acid site in the channel. Zhang et al. [26] reported a Zn/P/ZSM-5 core–shell structure catalyst with modified SiO_2_ that had a large number of strong acid sites in the core and a small amount of weak acid sites in the shell. The selectivity of PX in xylene isomers increased from the usual 23–24% to 89.6%. The deposition of SiO_2_ improved the selectivity of PX at the cost of reducing the aromatics content. The lifetime of the catalyst was also very short, only about 1 h. Li et al. [27] investigated the synergistic effects of Zn and P modifications on the pore structure and acidity of HZSM-5. They found that the introduction of P and Zn significantly reduced the acid strength of HZSM-5, increased the Lewis/Brönsted ratio, facilitated the aromatization of methanol while inhibiting the hydrogen transfer reaction. Additionally, doping P also enlarged the pore size of HZSM-5 and enhanced its coke resistance. Overloading Zn and P could affect the aromatic-selectivity; however, the optimal loading amounts were approximately 1% and 2%, respectively. Zhu’s research team [28,29] developed a mesoporous Zn-Mg-P/ZSM-5 catalyst for the direct conversion of methanol to para-xylene. The introduction of Zn enhanced the aromatization of methanol. Furthermore, the subsequent Mg-P co-modification improved the shape-selectivity of PX and promoted the production of light olefins. Jia et al. [30] prepared a Zn-P/HZSM-5 catalyst by modifying HZSM-5 with ZnSiF_6_·6H_2_O and H_3_PO_4_ solutions. The introduction of P and Zn facilitated the formation of benzene, toluene, and xylene (BTX) hydrocarbons. In addition, SiF_6_^2−^ efficiently removed the acidity on the outer surface of HZSM-5 catalyst, significantly enhancing its stability. Pan et al. [31] prepared an H[Zn, Al]ZSM-5/SiO_2_ composite catalyst by directly introducing Zn into the ZSM-5 framework through isomorphous substitution, followed by depositing SiO_2_ on the surface of the catalyst. The methanol conversion was almost 100%, PX-selectivity and PX yield were 95.6% and 18.2%, respectively. Table 1 summarizes the performance of typical modified ZSM-5 catalysts for MTA in the literature.

Phosphorus is commonly used to improve the selectivity of PX in MTA, but most modifications employ phosphoric acid as the phosphorus source. In our previous work [33], we found that the phosphoric-acid-modified HZSM-5 zeolite reduced the external surface area, pore size, and Brønsted acid sites, which considerably improved the selectivity of PX in X and xylene in aromatics, and the selectivity was enhanced with the increase in P content. The selectivity of PX reached nearly 90% when P content was 5%. However, the activity and aromatic selectivity of the catalyst were also substantially reduced due to the severe dealumination of the ZSM-5 framework caused by the overacidity of the phosphoric acid used in the modification process. The yield of PX on the 5% P/ZSM-5 catalyst was only 5.6%. In addition, we found that the introduction of Zn into ZSM-5 formed Zn-Lewis acid sites, which can promote the dehydrogenation of light hydrocarbons to aromatics and inhibit the hydrogen transfer reaction by depressing the Brønsted acidity, thus improving the selectivity of aromatics in the MTA reaction. However, the selectivity of PX in X hardly changed [5,9]. In this work, first of all, we hoped to find a P-containing modifier that is better than phosphoric acid in terms of catalyst activity, stability, and PX selectivity. The influence of different phosphorus sources including phosphoric acid (H_3_PO_4_), diammonium hydrogen phosphate ((NH_4_)_2_HPO_4_), and ammonium phosphate ((NH_4_)_3_PO_4_) on the performance of MTA was investigated. The effects of different phosphorus sources on the textural and acidic properties of ZSM-5 were significantly different, resulting in different catalytic properties. Subsequently, the Zn-P/ZSM-5 catalyst co-modified by Zn and P was prepared by introducing (NH_4_)_2_HPO_4_ with better modification effect into Zn/ZSM-5. The addition of Zn in the catalyst system can form a new aromatization active-center Zn-Lewis acid, so as to improve the selectivity of aromatics as much as possible while enhancing the selectivity of PX. The effects of P and Zn content on the selectivity and yield of MTA reaction was studied. The introduction of appropriate amounts of Zn and P species into ZSM-5 zeolite can play a synergistic role and increase the yield of PX in MTA.

## 2. Results and Discussion

### 2.1. Design of P/ZSM-5 Systems from Different Phosphorus Sources

#### 2.1.1. Synthesis and Characterization of P/ZSM-5 Systems

In this section, the influence of different phosphorus sources such as H_3_PO_4_, (NH_4_)_2_HPO_4_, and (NH_4_)_3_PO_4_ on the performance of MTA reaction and the physicochemical properties of ZSM-5 zeolites was compared. The catalysts modified by these different phosphorus sources had a P-loading content of 3% and were denoted as 3P-H_3_PO_4_, 3P-(NH_4_)_2_HPO_4_, and 3P-(NH_4_)3PO_4_, respectively.

**Figure 1:**
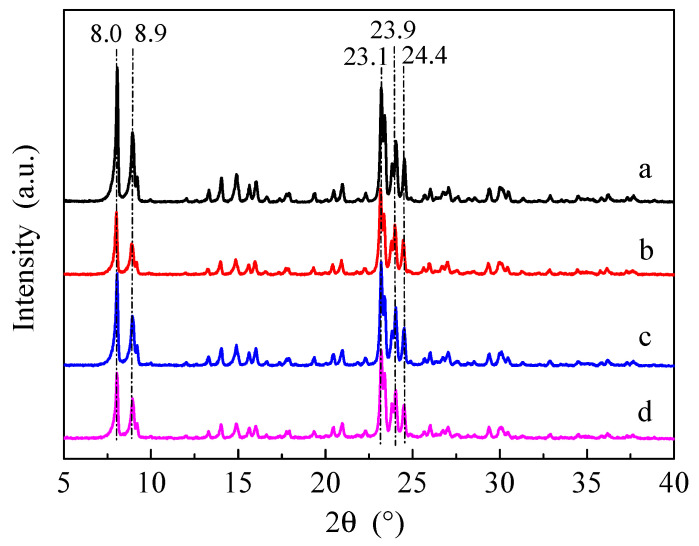
XRD patterns of the HZSM-5 and P/ZSM-5 zeolites: (**a**) HZSM-5, (**b**) 3P-H_3_PO_4_, (**c**) 3P-(NH_4_)_2_HPO_4_, and (**d**) 3P-(NH_4_)_3_PO_4_.

Figure 1 shows the X-ray powder diffraction (XRD) patterns of the HZSM-5 and P/ZSM-5 zeolites prepared using different phosphorus sources. All the samples exhibited similar characteristic diffraction peaks occurring at 2θ of 8.0°, 8.9°, 23.1°, 23.9° and 24.4° of MFI structure, suggesting that the introduction of phosphorus by impregnation had little impact on the structure of ZSM-5 framework [3,34]. No new diffraction peak was observed, indicating that the introduced P species were highly dispersed in the parent HZSM-5. Compared with HZSM-5, the diffraction peak intensity of P/ZSM-5 decreased slightly, which was probably due to the framework defects caused by a certain degree of dealumination of the ZSM-5 framework [25,32]. When (NH_4_)_2_HPO_4_ was introduced as a phosphorus source, the peak intensity decreased the least. Compared with the parent HZSM-5 zeolite, the relative crystallinity of 3P-H_3_PO_4_, 3P-(NH_4_)_2_HPO_4_, and 3P-(NH_4_)_3_PO_4_ was 82.5%, 90.2%, and 80.9%, respectively.

**Figure 2:**
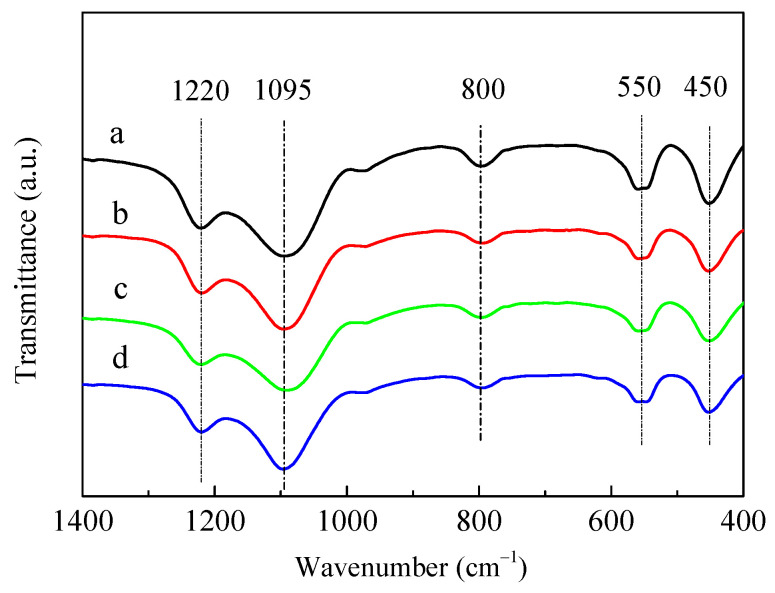
FT-IR spectra of HZSM-5 and P/ZSM-5 zeolites: (**a**) HZSM-5, (**b**) 3P-H_3_PO_4_, (**c**) 3P-(NH_4_)_2_HPO_4_, and (**d**) 3P-(NH_4_)_3_PO_4_.

Figure 2 shows the framework structures of the HZSM-5 and P/ZSM-5 catalysts characterized by Fourier-transform infrared (FT-IR) absorption spectroscopy. The characteristic IR bands of ZSM-5 zeolite with wavenumbers of 450, 550, 800, 1095, and 1220 cm^−1^ appeared [30,31]. All the parent HZSM-5 and P-modified ZSM-5 samples showed similar structure-sensitive bands at the same position. The absence of new IR band and obvious shift suggested that all samples possessed similar ZSM-5 framework structures and the introduced P species did not destroy the zeolite framework structures and get into the zeolite framework position [22,30]. This result was well matched with the XRD result shown in Figure 1. Jia et al. [30] proposed that partial zeolitic Si(OH)Al could interact with phosphate and be destroyed along with the formation of Si–OH, which resulted in an increase in the full width at half maximum at 1100 cm^−1^ for the P/HZSM-5 catalyst. No new band and significant shift were observed in P/HZSM-5.

**Figure 3:**
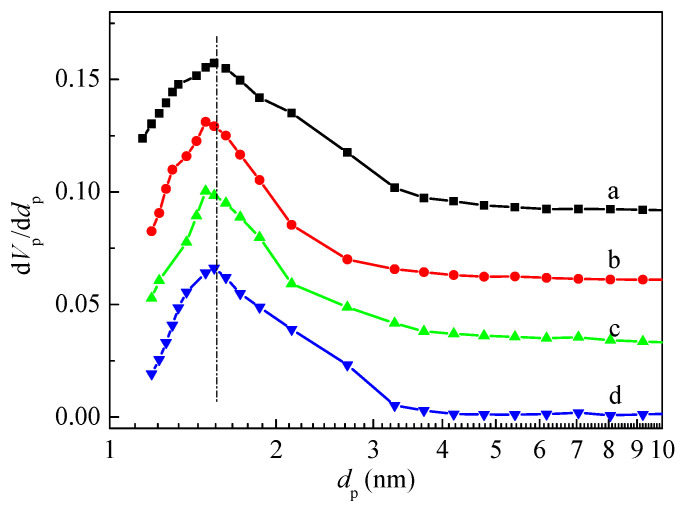
Pore size distribution of HZSM-5 and P/ZSM-5 zeolites: (**a**) HZSM-5, (**b**) 3P-H_3_PO_4_, (**c**) 3P-(NH_4_)_2_HPO_4_, and (**d**) 3P-(NH_4_)_3_PO_4_.

The Barrett-Joyner-Halenda (BJH) method is one algorithm model typically used to determine the pore size distribution [35]. Figure 3 showed the pore size distribution curves of HZSM-5 and P/ZSM-5 zeolites calculated by the BJH method using an adsorption branch. The porous structure of the P/ZSM-5 zeolites was related to the introduced phosphorus source. Compared with the parent HZSM-5, the impregnation of H_3_PO_4_ and (NH_4_)_2_HPO_4_ significantly narrowed the pore size distribution and slightly reduced the pore size. However, the effect when impregnated with (NH_4_)_3_PO_4_ on changing pore size distribution was relatively poor. The narrowing of pore size for P/ZSM-5 zeolites may be ascribed to the introduced P species located in the porous channel, which also reduced the accessibility of micropores, leading to the decrease in microporous volume [36] (Table 2).

**Figure 4:**
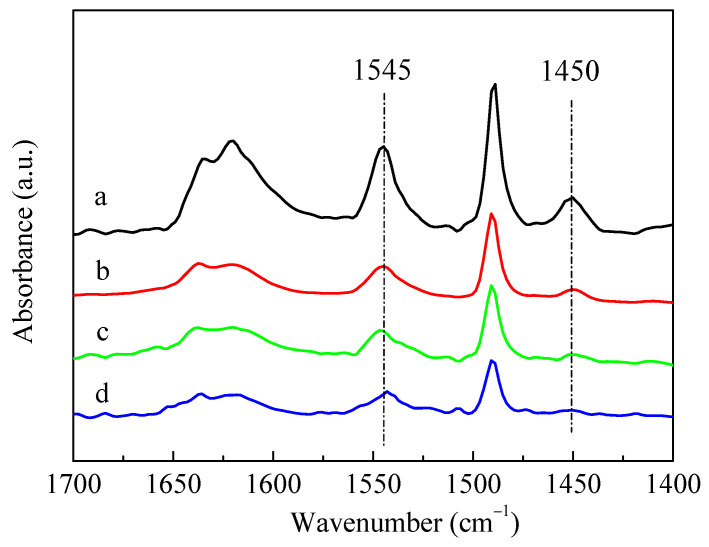
IR spectra of pyridine adsorption on HZSM-5 and P/ZSM-5 zeolites: (**a**) HZSM-5, (**b**) 3P-H_3_PO_4_, (**c**) 3P-(NH_4_)_2_HPO_4_ and (**d**) 3P-(NH_4_)_3_PO_4_.

The infrared spectra for pyridine adsorption (Py-IR) results of HZSM-5 and P-modified ZSM-5 zeolites with different phosphorus sources were given in Figure 4. The adsorption of pyridine on HZSM-5 resulted in the conversion of Brønsted (B) acid sites into pyridine ions with the infrared absorption band of 1545 cm^−1^, and the weaker band at 1450 cm^−1^ was assigned to the Lewis (L) acid sites due to the dehydroxylation of the ZSM-5 zeolite [37]. The introduction of different P sources all led to a decrease in the characteristic peaks of B acids and L acids of ZSM-5 zeolites, but the degree of reduction varied. Meanwhile, L acids decreased more than B acids, which can be deduced by observing the ratio of L acids to B acids (L/B), as summarized in Table 2. The reduction in B acids and L acids was mainly due to the impregnated phosphorus species covering the acid centers [28]. Among these samples, the characteristic peaks of B acids and L acids decreased the most after loading (NH_4_)_3_PO_4_. It can also be seen from Table 2 that the amounts of B acids and L acids of the P-modified ZSM-5 zeolites had little difference after the impregnation of H_3_PO_4_ and (NH_4_)_2_HPO_4_, while the amounts of B acids and L acids of 3P-(NH_4_)_3_PO_4_ were only about half of those of 3P-H_3_PO_4_ and 3P-(NH_4_)_2_HPO_4_. Nitrogen adsorption–desorption results showed that, compared with the unmodified HZSM-5 zeolite, the specific surface area, external surface area, total pore volume, and micropore volume of P-modified ZSM-5 zeolites all decreased significantly after impregnation with different phosphates (Table 2). Similar to the results of Py-IR, compared with the samples of 3P-H_3_PO_4_ and 3P-(NH_4_)_2_HPO_4_, the specific surface area and pore volume of 3P-(NH_4_)_3_PO_4_ decreased to a greater extent. When (NH_4_)_2_HPO_4_ was used as a P source, the specific surface area and pore volume of the modified 3P-(NH_4_)_2_HPO_4_ sample were the largest, while the external specific surface area was the smallest. This was possibly attributed to the fact that when (NH_4_)_2_HPO_4_ was introduced as a P source, more P species were gathered on the surface of the ZSM-5 zeolite and fewer entered the channel than the other two P sources.

**Table 2:** Physicochemical properties of HZSM-5 and P/ZSM-5 zeolites.

Samples	Si/Al	B Acids (mmol·g^−1^)	L Acids (mmol·g^−1^)	L/B	S_B_ (m^2^·g^−1^)	S_e_ (m^2^·g^−1^)	V_t_ (cm^3^·g^−1^)	V_mic_ (cm^3^·g^−1^)	Microporosity (%)
HZSM-5	36	0.170	0.054	0.32	381	13	0.21	0.18	85.7
3P-H_3_PO_4_	36	0.073	0.009	0.12	285	8	0.16	0.13	81.2
3P-(NH_4_)_2_HPO_4_	36	0.067	0.008	0.12	290	8	0.17	0.15	88.2
3P-(NH_4_)_3_PO_4_	37	0.036	0.005	0.14	225	9	0.13	0.11	84.6

Note: Si/Al atomic ratio (Si/Al) obtained by ICP-AES analysis; density of B acids and L acids determined by Py-IR and calculated by following the procedures reported by Madeira et al. [38]; L/B, the ratio of the amounts of L acids to that of B acids; S_B_, BET surface area; S_e_, external surface area, calculated by the t-plot method; V_t_, total volume; V_mic_, micropore volume, obtained by the t-plot method; microporosity, the ratio of micropore volume to total pore volume.

**Figure 5:**
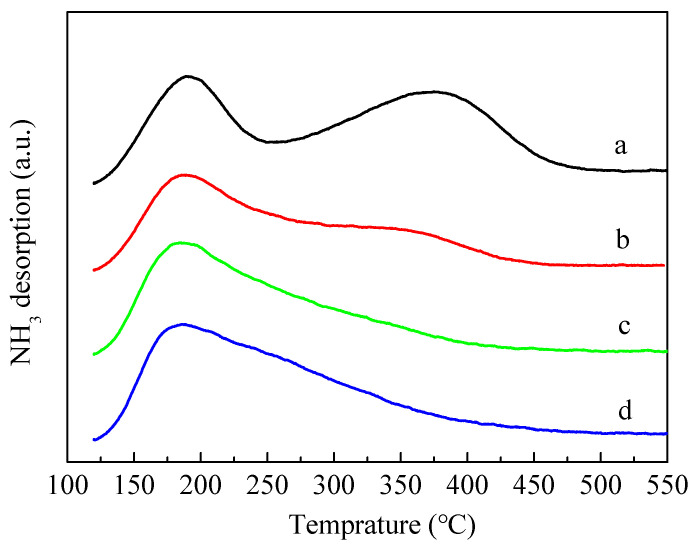
NH_3_-TPD profiles of the parent HZSM-5 and P-modified ZSM-5 zeolites: (**a**) HZSM-5, (**b**) 3P-H_3_PO_4_, (**c**) 3P-(NH_4_)_2_HPO_4_, and (**d**) 3P-(NH_4_)_3_PO_4_.

Figure 5 shows the results of temperature-programmed desorption of NH_3_ (NH_3_-TPD). The introduction of P obviously caused the area of the high temperature peak near 350–400 °C to decrease and the corresponding temperature of the high temperature peak shifted towards the lower temperature, indicating that the introduction of P led to a decline in the number and intensity of strong acid sites in ZSM-5 zeolites. It is also evident from the figure that the high temperature peaks of 3P-(NH_4_)_2_HPO_4_ and 3P-(NH_4_)_3_PO_4_ samples almost disappeared. Compared with 3P-(NH_4_)_2_HPO_4_ and 3P-(NH_4_)_3_PO_4_, the strong acids of 3P-H_3_PO_4_ also decreased significantly, but the decrease was relatively minimal.

**Figure 6:**
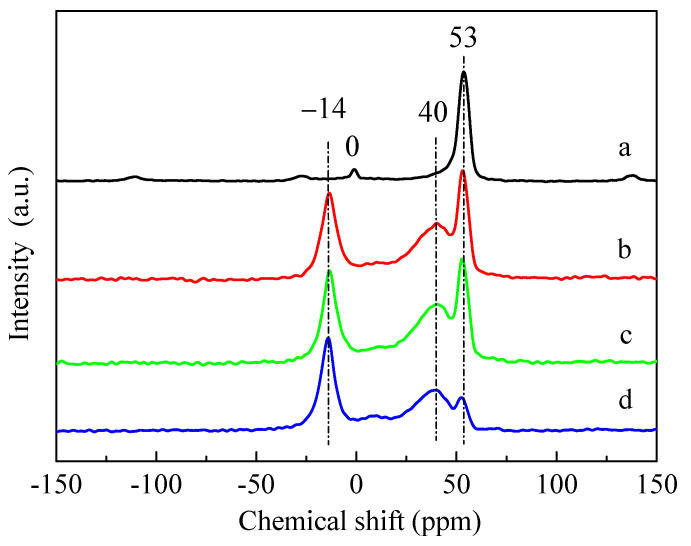
^27^Al MAS NMR spectra of the parent HZSM-5 and P-modified ZSM-5 zeolites: (**a**) HZSM-5, (**b**) 3P-H_3_PO_4_, (**c**) 3P-(NH_4_)_2_HPO_4_, and (**d**) 3P-(NH_4_)_3_PO_4_.

It can be seen from the ^27^Al magic angle spinning nuclear magnetic resonance (^27^Al MAS NMR) spectra of Figure 6 that, for the HZSM-5 sample, there were mainly a characteristic peak of typical tetrahedral framework aluminum (53 ppm) and a relatively weak peak of octahedral aluminum caused by extra-framework aluminum species (0 ppm) [33,39]. After the introduction of P, the peak of tetrahedral framework aluminum at 53 ppm decreased, indicating that the introduction of P resulted in the dealumination of the HZSM-5 framework structure, and a new peak of dealumination was obviously generated at 40 ppm due to the destruction of framework aluminum. The disappearance of the 0 ppm peak in the HZSM-5 zeolite was due to the combination of the introduced P species with extra-framework Al species, and a new peak was generated correspondingly at −14 ppm [40,41]. The ^27^Al MAS NMR results showed that the dealumination of the HZSM-5 zeolite framework occurred in the process of introducing different P sources, and the most serious dealumination occurred when (NH_4_)_3_PO_4_ was used as a P source, consistent with the XRD, pore volume, and Py-IR results. Although the dealumination of ZSM-5 zeolite framework occurred in the process of impregnating different phosphorus sources. As the impregnated catalysts were directly dried and calcined without washing treatment, the Si/Al ratios of P-modified ZSM-5 catalysts measured by ICP were basically unchanged.

In summary, the effects of different P sources on the specific surface area, pore volume, acidity, and other properties of HZSM-5 zeolite were varied. The use of (NH_4_)_3_PO_4_ as a P source resulted in the most severe dealumination and weakest acidity among the samples. Additionally, the reduction in the specific surface area and pore volume of 3P-(NH_4_)_3_PO_4_ was also the largest. When H_3_PO_4_ was used as a P source, the strong acid sites, B acid sites, and L acid sites of 3P-H_3_PO_4_ decreased the least. However, it was possible that the P species introduced by (NH_4_)_2_HPO_4_ gathered more on the surface of the zeolite than those entering the channels, resulting in the highest specific surface area and pore volume, and the smallest external surface area.

#### 2.1.2. Catalytic Performance of P/ZSM-5 in MTA

**Figure 7:**
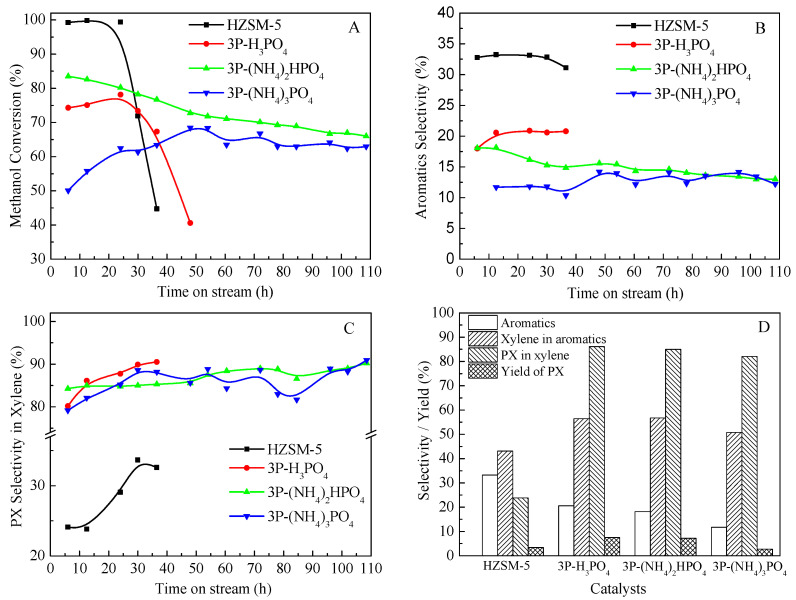
Methanol conversion (**A**), aromatics selectivity (**B**), selectivity of PX in xylene (**C**) of MTA with the time on stream (TOS) and product selectivity or yield at a TOS of 12.5 h (**D**) over HZSM-5 and P/ZSM-5 catalysts.

The catalytic performances of HZSM-5 and P/ZSM-5 zeolites prepared by different phosphorus sources in MTA were illustrated in Figure 7. As shown in Figure 7A, the addition of different P sources all led to a decrease in the activity of HZSM-5 zeolite catalyst, and the conversion of methanol decreased to less than 90%. However, the change trend of the stability and conversion of the catalysts after impregnation with different P sources was obviously different. The methanol conversion of 3P-(NH_4_)_2_HPO_4_ catalyst decreased the least, and its stability was significantly better than that of the 3P-H_3_PO_4_ catalyst. The 3P-(NH_4_)_3_PO_4_ catalyst had the lowest methanol conversion, with an initial methanol conversion of only about 50%, which increased slightly over time on stream and tended to be stable for a period of time. It was suggested that the impregnation of phosphorus could enhance the stability of the framework Al of ZSM-5 zeolite during the catalytic reaction, and thus improve the stability of the catalyst [42,43]. The different P-source-modified ZSM-5 zeolites exhibited great differences in the activity and stability of MTA, which may be due to the factors such as the acidity and pore volume. The severe dealumination of the zeolite framework caused by the introduction of (NH_4_)_3_PO_4_ led to the most significant decrease in its activity, while the P species introduced by (NH_4_)_2_HPO_4_ were more concentrated on the surface of the zeolite, and less entered the pores, resulting in the largest specific surface area, total pore volume, micropore volume, and microporosity. B acid sites in a zeolite are known as MTA catalytic centers. The methanol is dehydrated in the presence of B acids to form dimethyl ether, which is converted to hydrocarbons by further dehydration and other reactions. The introduction of P significantly reduced B acids in ZSM-5 zeolite, resulting in a decrease in catalyst conversion. The methanol conversion of 3P-(NH_4_)_3_PO_4_ catalyst with the most severe B acid decline was the lowest. Jia et al. [30] also found that after H_3_PO_4_ modification, the catalytic activity of P/ZSM-5 zeolite was significantly decreased, and the initial conversion rate of methanol was reduced to less than 80%. Moreover, the microporosity also had an effect on catalytic activity. For conventional microporous zeolites, most of the acid sites were located inside the zeolitic micropores [44]. Bibby et al. [45] reported that the deactivation of ZSM-5 zeolite was mainly due to the direct coke deposition on the active acid sites and indirectly by blocking diffusion into other micropores. Unless the pore entrances were completely covered, the reactants could gain access to the active sites. Kim et al. [46] found a similar result in that the zeolite catalyst continued to be active as long as internal micropores were accessible. In this study, the acidity and external surface area of 3P-H_3_PO_4_ and 3P-(NH_4_)_2_HPO_4_ samples were similar, but the catalytic stability of 3P-(NH_4_)_2_HPO_4_ was significantly better than that of 3P-H_3_PO_4_. It was probable that 3P-(NH_4_)_2_HPO_4_, with higher microporosity, provided more active sites for access, thereby exhibiting a greater ability to resist coke deposition. Meanwhile, compared with the parent HZSM-5, due to the reduction of the concentration of strong acid sites in HZSM-5, the formation rate of internal coke was decreased, thus enhancing the catalytic stability. Figure 7B,C gave the aromatic selectivity and PX selectivity in xylene with the time on stream for MTA, respectively. The different P-source-modified ZSM-5 had a significantly reduced aromatic selectivity, following the sequence of HZSM-5 > 3P-H_3_PO_4_ > 3P-(NH_4_)_2_HPO_4_ > 3P-(NH_4_)_3_PO_4_. Moreover, the introduction of P to ZSM-5 zeolite significantly improved the selectivity of PX in X, with PX selectivity reaching above 80%. The selectivity of xylene in aromatics and PX in X on 3P-H_3_PO_4_ and 3P-(NH_4_)_2_HPO_4_ were over 55% and 85%, respectively. The PX yield of HZSM-5, 3P-H_3_PO_4_, 3P-(NH_4_)_2_HPO_4_, and 3P-(NH_4_)_3_PO_4_. was 3.4%, 7.5%, 7.2%, and 2.7%, respectively (Figure 7D). After the modification of H_3_PO_4_ and (NH_4_)_2_HPO_4_, the yield of PX could be more than doubled, while the modification effect of (NH_4_)_3_PO_4_ decreased the yield of PX. It was also found that the introduction of P into ZSM-5 reduced the activity of the catalyst for other reactions. In the C_4_-olefin-cracking reaction, the conversion of C_4_ decreased sharply with the increase in P content, which was likely be due to a reduction in the density of acid sites and a decrease in surface area and pore volume [47]. Ghiaci et al. [42] also found that the P-modified ZSM-5 catalyst could significantly improve the selectivity of PX in the methylation reaction of toluene. However, as part of the P species entered the pore of the zeolite and covered a part of the strong acid sites, there was a resulting significant decrease in the activity.

As mentioned above, the introduction of P has significant effects on the framework structure, pore structure and acidity of the ZSM-5 zeolite, such as a decrease in pore volume, specific surface area, acid strength, and acid amount. This indicates that in the P-modified ZSM-5 zeolite, P species enter the channel of the zeolite, which not only causes the blockage of the catalyst pore, but also covers the surface acid site, reducing the strong acid center and decreasing the catalytic activity. On the other hand, it is the narrowing of pore size and the covering of the surface acid site that improve the shape selectivity of para-xylene in the MTA process and increase the selectivity of PX in X from approximately 24% to more than 80%. The P source has a significant influence on the activity, stability, and PX selectivity of the ZSM-5 catalyst in the MTA reaction. In terms of catalyst stability and PX selectivity, (NH_4_)_2_HPO_4_, as a P source, exhibits a greater modification effect.

### 2.2. Design of Zn-P/ZSM-5 Systems

#### 2.2.1. Synthesis and Characterization of Zn-P/ZSM-5 Systems

The introduction of P can significantly enhance the selectivity of PX in xylene, as well as reduce the aromatics selectivity. In the previous work, we found that the introduction of Zn into the ZSM-5 zeolite can effectively improve the methanol aromatization activity of MTA [5,9,48]. Therefore, we tried to introduce Zn (zinc nitrate) and P (diammonium hydrogen phosphate) into the ZSM-5 zeolite, expecting that the two elements would have a synergistic modification effect, so as to obtain an excellent catalyst with high aromatics selectivity and high PX selectivity. The Zn- and P-modified ZSM-5 catalysts in this section are labeled xP-yZn, where x and y represent the mass percentage of P and Zn elements introduced, respectively.

**Figure 8:**
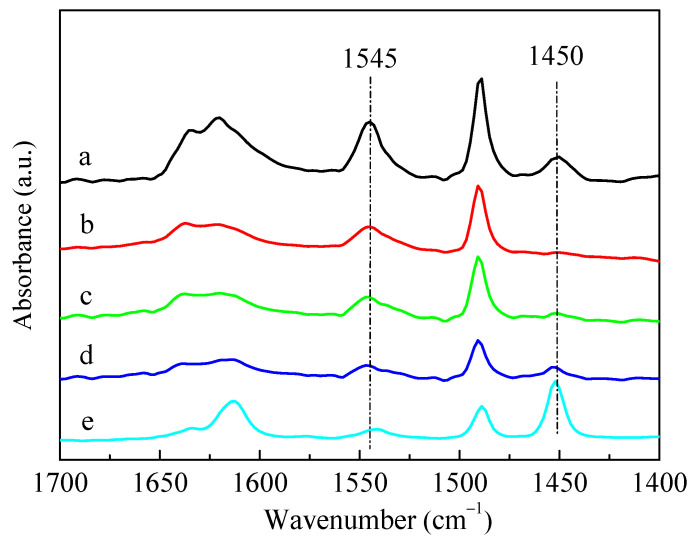
IR spectra of pyridine adsorption for HZSM-5 and ZSM-5 zeolites modified by Zn and P: (**a**) HZSM-5, (**b**) 5P, (**c**) 5P-1Zn, (**d**) 5P-3Zn, and (**e**) 5P-10Zn.

As shown in Figure 8, the introduction of P significantly reduced the B acid (1545 cm^−1^) and L acid sites (1450 cm^−1^) of the HZSM-5 zeolite. This phenomenon was primarily due to the impregnated phosphorus species covering the acid sites of ZSM-5 zeolite [29]. It was reported that partial P species could chemically bond with L acid sites to form the distorted AlO_4_ tetrahedron, and interact with the partial Si(OH)Al of B acid sites to form Si–OH [30,47]. Blasco et al. [36] proposed that phosphorus was mainly present in the form of cationic pyrophosphate, partially neutralizing the B acids of ZSM-5 zeolite. When the HZSM-5 zeolite was modified by Zn and P, the B acid sites further decreased with the increase in Zn-loading, while the L acid sites gradually increased. The increase in L acid sites was due to the combination of the introduced Zn with the B acid sites of the parent HZSM-5 to form a new Zn-Lewis (Zn-L) acid sites, which was also the active center of aromatization [49]. In previous work [48], we confirmed that most of the Zn introduced via impregnation existed in the form of Zn-Lewis species (ZnOH^+^) generated from zinc species interacted with the proton acid sites, and a small amount of ZnO was also formed and dispersed in the zeolite channel.

#### 2.2.2. Catalytic Performance of Zn-P/ZSM-5 in MTA

**Figure 9:**
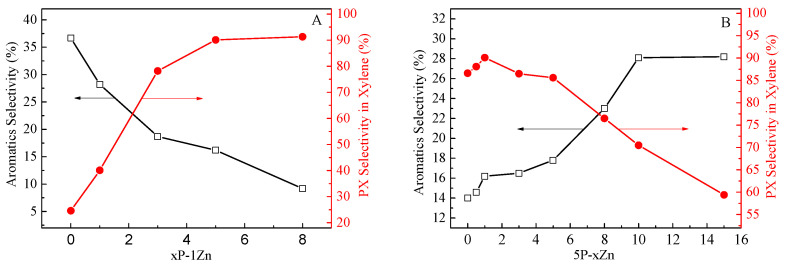
The selectivity of aromatics (**A**) and PX-selectivity in xylene (**B**) over HZSM-5 and Zn-P/ZSM-5 zeolites with different Zn- and P-loading contents in MTA. The selectivity data were obtained at 12.5 h on stream for MTA.

Figure 9 and Table 3 show the catalytic performance of the HZSM-5 and Zn-P/ZSM-5 zeolites with different Zn- and P-loading contents in MTA. As listed in Table 3, the loading of Zn on HZSM-5 zeolite obviously increased the aromatics selectivity, while the selectivity of C_1_-C_4_ alkane significantly decreased, indicating that the introduction of Zn into HZSM-5 can inhibit the hydrogen transfer reaction, reduce the generation of low-carbon alkane, and improve the aromatics selectivity. The increase in C_2_-C_5_ olefins suggests that the olefins could be generated either via a cracking reaction on B acid sites or via dehydrogenation of the Zn-L acid sites [50]. It can be clearly seen from Figure 9A that when the loading of Zn was constant, the PX selectivity in xylene gradually increased with the increase in the second-element P-loading. When the P content was increased to more than 5%, the PX selectivity in the xylene could reach more than 90%. The introduction of P can improve the selectivity of PX in xylene, but at the cost of reducing the aromatics selectivity and methanol conversion. Both the aromatics selectivity and methanol conversion decreased significantly with the increase in P-loading. When P-loading was 8%, the aromatics selectivity was less than 10% and methanol conversion was only about 50%. Although the selectivity of PX in xylene was as high as 91.3%, the yield of PX was only 2.3%, which was less than that of the unmodified HZSM-5. Meanwhile, the increase in P-loading greatly improved the selectivity of light olefin (C_2_-C_5_), which was mainly attributed to the sharp decrease in acidity, resulting in the inhibition of the hydrogen transfer reaction [28]. When the loading of P was fixed at 5% and the impregnation content of Zn was gradually increased, the selectivity to aromatics and methanol conversion were gradually improved, while the selectivity to PX in xylene increased slowly at first and then decreased (Figure 9B). The maximum selectivity of PX in xylene was 90.1% when the loading of P and Zn were 5% and 1%, respectively. Compared with the 5P catalyst loaded with only (NH_4_)_2_HPO_4_, followed by loading 1% Zn, the methanol conversion and aromatics selectivity increased by 26.1% and 13.6%, respectively. Meanwhile, the PX selectivity of 5P-1Zn catalyst slightly increased from 86.6% to 90.1%, and the PX yield increased by 59%. Moreover, the yield of PX also increased first and then decreased with the increase in Zn-loading, when the loading of P and Zn were 5% and 10%, respectively, the yield of PX was the highest (10.3%).

Although the introduction of P into the ZSM-5 zeolite via impregnation can improve the PX selectivity, the aromatization activity of the catalyst was inevitably reduced due to the serious coverage of the active sites by the introduced P species and the resulting weakened acidity and mass transfer restriction, which indicates that the aromatics selectivity and methanol conversion were seriously decreased. The addition of the second modified element Zn could improve the aromatics selectivity to a certain extent without reducing the selectivity of PX in xylene.

For the MTA reaction, methanol is first dehydrated to form dimethyl ether under the action of B acids, and then to generate ethene, propene, and higher olefins’ hydrocarbon pool mechanism [51,52]. The olefins undergo complex reaction processes such as oligomerization, methylation, cracking, cyclization, hydrogen transfer, and dehydrogenation to generate aliphatic hydrocarbons, light alkanes, and aromatics [29,33,48]. In the present work, when doping P for modification, the introduced P species may cover or interact with B acids and L acids, resulting in a significant reduction in the density of B acids and L acids [42,53]. The addition of P had a passivating effect on the acidity of the catalyst, especially on B acids, resulting in the decrease in methanol conversion and aromatics selectivity [54]. The advantage was that, with the decrease in the concentration of strong acid sites in the HZSM-5 zeolite, the formation rate of internal coke decreased, thus enhancing the catalytic stability. Otherwise, the introduction of P species also narrowed the pores and reduced the external surface area of the ZSM-5 zeolite, which was more conducive to the formation of PX product in MTA. On the one hand, the narrowing of the pores limited the formation of o-xylene and m-xylene in the xylene isomers and the diffusion of these two xylene isomers out of the zeolite channels. On the other hand, due to the large amount of surface acidic sites covered, it was difficult for the generated PX to continue to undergo alkylation and isomerization reactions on the surface of the ZSM-5 zeolite. It was validated by Zhang and coworkers that the external acidic sites determined the isomerization of p-xylene [26]. The introduction of Zn reduced the B acid sites and decreased the formation of alkanes by inhibiting the hydrogen-transfer reaction. In addition, the generated Zn-Lewis acids that located on B acid sites were conducive to the dehydrogenation of olefins to the formation of aromatics. The formation of aromatics was related to both the B acids and the Zn-Lewis acids. The introduction of an appropriate amount of P species into Zn/ZSM-5 zeolite can play a synergistic role with Zn species to increase the yield of PX in MTA.

## 3. Materials and Methods

### 3.1. Catalyst Preparation

The parent HZSM-5 zeolite catalyst was prepared exactly according to the procedures in our previous work [33]. Incipient wetness impregnation was used to introduce P or Zn into the HZSM-5 zeolite and the different P or Zn content was obtained by changing the concentration of (NH_4_)_2_HPO_4_ or Zn(NO_3_)_2_ solution, then dried at 100 °C overnight and calcined at 560 °C for 6 h in air. The Zn-P/ZSM-5 zeolite was obtained by impregnating the Zn(NO_3_)_2_ solution first, after drying and calcining, and then impregnating the (NH_4_)_2_HPO_4_ solution, and drying and calcining. The catalyst was denoted by xP-yZn, where x and y represent the mass percentage of P and Zn elements introduced, respectively.

### 3.2. Catalyst Characterization

Powder X-ray diffraction (XRD) patterns were collected on a Rigaku MiniFlex II X-ray diffractometer (Rigaku, Tokyo, Japan) with Cu Kα radiation (30 kV and 15 mA) at a scanning speed of 4°/min. The relative crystallinity of the P/ZSM-5 catalyst was estimated by comparing the total peak area in the range of 2θ from 7° to 10° and 22° to 25° with that of the parent HZSM-5. The specific surface area, pore size distribution, and pore volume were measured at 77 K on a BELSORP-max gas adsorption analyzer (MicrotracBEL, Osaka, Japan). The actual amount of zinc element in the Zn/ZSM-5 zeolites was determined by inductively-coupled plasma atomic emission spectroscopy (ICP-AES, Autoscan16, TJA, Boston, MA, USA). The ^27^Al magic angle spinning nuclear magnetic resonance (^27^Al MAS NMR) spectra were recorded on Bruker Avance III 600 spectrometer (Bruker, Karlsruhe, Germany) with 1.0 M aqueous solution of Al(NO_3_)_3_ as the reference at 104.3 MHz. Infrared spectra for pyridine adsorption (Py-IR, Tensor 27 FT-IR spectrometer, Bruker, Karlsruhe, Germany) and temperature-programmed desorption of NH_3_ (NH_3_-TPD, AutoChem II 2920 chemisorption analyzer, Micromeritics, Atlanta, GA, USA) were performed by following similar procedures with the same instrument as reported previously [33,48].

### 3.3. Catalyst Tests

The MTA reaction was carried out in a fixed-bed reactor (Pengxiang, Tianjin, China) with methanol weight hourly space velocity (WHSV) of 3.2 h^−1^ at 390 °C and 0.5 MPa. The catalyst was pressed and sieved into 20–40 mesh and pre-treated for 8 h at 400 °C in a N_2_ flow before reaction. The reaction products were separated using a cold trap and then analyzed via gas chromatography (GC 7890A, Agilent, Palo Alto, CA, USA).

The methanol conversion and product-selectivity were calculated with the following equations:

(1) $$Methanol\;conversion\;\left(\%\right)=\frac{n_{CH_{3}OH}^{i}-n_{CH_{3}OH}^{o}}{n_{CH_{3}OH}^{i}}\times 100,$$

(2) $$Product\;selectivity\;\left(\%\right)=\frac{m_{i}}{m}\times 100,$$

where $n_{CH_{3}OH}^{i}$, $n_{CH_{3}OH}^{o}$, $m_{i}$, and m represent the total amount of methanol feed, the amount of unreacted methanol, the mass of product *i* and the total mass of all hydrocarbons products, respectively.

In addition, the selectivity of PX in xylene refers to the ratio of PX to the three xylene isomers.

## 4. Conclusions

The introduction of P led to the covering of the active acid sites and the narrowing of the pore of the ZSM-5 zeolite, which improved the shape-selectivity of PX in the MTA reaction. The P source had a significant influence on the catalyst activity, stability, and PX-selectivity in xylene. Among the three selected modifiers, H_3_PO_4_, (NH_4_)_2_HPO_4_, and (NH_4_)_3_PO_4_, the ZSM-5 zeolite modified by (NH_4_)_2_HPO_4_ exhibited better catalyst stability and PX-selectivity due to its larger specific surface area, pore volume, and suitable acidity. The most serious dealumination of the modified ZSM-5 zeolite occurred when (NH_4_)_3_PO_4_ was used as a P source, resulting in the smallest specific surface area, pore volume, and acidity of the catalyst, and thus, the worst activity.

The effects of Zn and P of the Zn-P/ZSM-5 zeolite on the aromatic-selectivity and PX-selectivity in xylene was almost the opposite. The selectivity of PX in xylene gradually increased with the increase in P-loading. When the P content was increased to more than 5%, the PX-selectivity in xylene could reach more than 90%. However, the aromatics selectivity and methanol conversion also decreased sharply, resulting in little improvement in PX yield. The loading of Zn could introduce Zn-Lewis acid sites to provide aromatization active centers and improved the aromatic-selectivity. However, excessive Zn decreased the selectivity of PX in xylene. The catalyst activity and aromatic-selectivity can be improved to some extent with an appropriate ratio of Zn and P, while the para-selectivity of xylene was maintained or increased. Compared with the 5P catalyst modified with only (NH_4_)_2_HPO_4_, the PX-selectivity of 5P-1Zn catalyst was slightly improved from 86.6% to 90.1%, and the PX yield was increased by 59%. The maximum yield of PX was 10.3% when the loading of P and Zn were 5% and 10%, respectively. The results of this work contribute to a greater understanding of the preparation of the catalyst for methanol conversion to para-xylene and the design of a catalyst with better performance.

## Figures and Tables

**Table 1:** Summary of typical modified ZSM-5 catalysts for MTA in the literatures.

Catalysts	Modification Method	Modifiers	Reaction Conditions		Modification Effect				Ref.
			Temp. (°C)	WHSV ^a^ (h^−1^)	Lifetime ^b^ (h)	S_Arom_ ^c^ (%)	PX/X (%)	PX Yield (%)	
H[Zn, Al]ZSM-5	Direct synthesis	Zn(NO_3_)_2_, ammonia	437	3.2	32/37	31.0/46.7 (BTX)	–	–	[8]
Zn/HZSM-5	Impregnation	Zn(NO_3_)_2_	390	3.2	170/80	36.2/45.8	23.8	–	[9]
Ga/HZSM-5	Alkaline treatment, impregnation	Ga(NO_3_)_3_	475	1.06	11.5/9.0	38.7/51.7	17.5	–	[12]
Zn/ZSM-5	Direct synthesis	ZnCl_2_	450	5	>5	41/56	–	–	[19]
Ga-ZSM-5	Direct synthesis, HCl treatment	Ga_2_O_3_	400	10	18/30	18.2/29.3 (C_6_, C_6+_)	–	–	[20]
Mg/Zn/Si/HZSM-5	Si-CLD ^d^, Zn, Mg-impregnation	PPMS ^e^, Zn(NO_3_)_2_, Mg(NO_3_)_2_	460	1.0	>12	38.4/57.3	98.9	21.24	[25]
Zn/P/Si/ZSM-5	Zn,P-impregnated, Si-CLD	Zn(NO_3_)_2_, H_3_PO_4_, TEOS	475	0.79	–	44.6/61.7	89.6	–	[26]
Zn-P/HZSM-5	Impregnation	Zn_3_(PO_4_)_2_, H_3_PO_4_	480	4.7	13	25/61.2	–	–	[27]
Zn-Mg-P/ZSM-5	Alkaline treatment, impregnation	Zn(NO_3_)_2_, Mg(NO_3_)_2_, H_3_PO_4_	400	2.4	50	35.9/43.6	90.8	19	[28]
Zn-P/HZSM-5	P-impregnation, Zn-ion exchange	H_3_PO_4_, ZnSiF_6_	400	0.7	462 ^f^	35.6/46.8 (BTX)	–	–	[30]
H[Zn,Al]ZSM-5/SiO_2_	Zn-direct synthesis, Si-CLD	Zn(NO_3_)_2_, TEOS	425	2.5	34/24	33.8/40.0	95.6	18.2	[31]
Zn/ZSM-5/silicalite-1	Zn-ion exchanged, Si-direct synthesis	Zn(NO_3_)_2_, fumed silica	400	0.74	–	–	99	40.7	[32]

^a^ Weight hourly space velocity; ^b^ catalyst lifetime over pristine and modified ZSM-5 catalysts; ^c^ aromatic-selectivity over pristine and modified ZSM-5 catalysts; ^d^ chemical liquid deposition; ^e^ Polyphenylmethylsiloxane; ^f^ methanol conversion: 70–75%.

**Table 3:** Product distribution of MTA reaction over HZSM-5 and Zn-P/ZSM-5 zeolites.

Catalysts	Conv._MeOH_ ^a^	Production-Selectivity (%)					Xylene in Aromatics	PX in X	Yield of PX ^c^
		C_1_^−^~C_4_^−^	C_2_^=^~C_5_^=^	C_5+_ Non-Aromatics	Aromatics	Others ^b^	-	-	-
HZSM-5	99.8	37.9	8.2	19.1	33.3	1.5	43.1	23.8	3.4
1Zn	99.5	28.1	14.9	18.6	36.7	1.7	43.0	24.4	3.8
1P-1Zn	98.8	18.9	32.0	20.1	28.2	0.8	45.7	40.1	5.1
3P-1Zn	83.8	14.0	46.4	19.9	18.7	1	55.6	78.2	6.8
5P-1Zn	77.8	11.7	57.1	14.5	15.9	0.8	60.7	90.1	6.8
8P-1Zn	51.2	9.7	71.6	9.2	9.2	0.3	52.4	91.3	2.3
5P	61.7	12.7	59.6	13.1	14.0	0.6	58.0	86.6	4.3
5P-0.5Zn	69.9	12.4	56.1	15.6	14.6	1.3	57.5	88.1	5.2
5P-1Zn	77.8	11.7	57.1	14.5	15.9	0.8	60.7	90.1	6.8
5P-3Zn	83.4	13.8	52.2	16.7	16.5	0.8	56.4	86.5	6.7
5P-5Zn	92.7	13.1	46.2	22.0	17.8	0.9	55.6	85.6	7.9
5P-8Zn	96.6	28.5	18.7	28.8	23.0	1	51.4	76.8	8.8
5P-10Zn	98.4	18.8	21.8	30.5	28.1	0.8	52.8	70.5	10.3
5P-15Zn	99.1	17.0	28.3	25.2	28.2	1.3	51.9	59.4	8.6

^a^ Conv._MeOH_, methanol conversion; ^b^ others, H_2_ and CO_X_; ^c^ the yield of PX is calculated through methanol conversion × aromatics selectivity × xylene selectivity in aromatics × PX selectivity in X. The data were obtained at 12.5 h on stream for MTA.

## Data Availability

Data are contained within the article.

## Sample Availability

Samples of the compounds are available from the authors upon request.
